# The effectiveness and safety of leflunomide in the treatment of giant cell arteritis: a systematic review and meta-analysis

**DOI:** 10.1093/rap/rkaf128

**Published:** 2025-11-07

**Authors:** Linda M Zhu, Arielle Mendel, Carolyn Ross, Jean-Paul Makhzoum

**Affiliations:** Internal Medicine, Hopital Sacre-Coeur, University of Montreal, Montreal, QC, Canada; Lupus and Vasculitis Clinic, McGill University Health Center, McGill University, Canadian Vasculitis Research Network, Montreal, QC, Canada; Vasculitis Clinic, Hopital Sacre-Coeur, University of Montreal, Canadian Vasculitis Research Network, Montreal, QC, Canada; Vasculitis Clinic, Hopital Sacre-Coeur, University of Montreal, Canadian Vasculitis Research Network, Montreal, QC, Canada

**Keywords:** leflunomide, giant cell arteritis, vasculitis, glucocorticoid

## Abstract

**Objectives:**

The objective of this systematic review is to assess the effectiveness and safety of leflunomide in the treatment of new-onset refractory or relapsing giant cell arteritis (GCA) as a glucocorticoid (GC)-sparing agent.

**Methods:**

We searched MEDLINE, Cochrane Library, Embase, clinical trial registries and other grey literature sources for randomized controlled trials, cohort studies, case-control studies and case series that reported on the use of leflunomide in GCA. The primary effectiveness outcome was the incidence (proportion) of patients who attained sustained GC-free remission at 6 to12 months, defined by the absence of signs or symptoms of GCA, and/or normalization of inflammatory markers, and/or radiologic response, plus complete discontinuation of GC. The secondary outcomes were remission on low-dose GC and adverse events. There was no available comparator. We performed a meta-analysis using a random effects model. Included studies were appraised for risk of bias.

**Results:**

Of 366 screened studies, 11 observational studies were included in the analysis, pooling data from 358 patients. The pooled proportion of patients achieving sustained GC-free remission was 45% (95% CI 25–64, *P* < 0.001), with high heterogeneity *I*^2^ test = 90.3% (*Q* = 70.65, *P* < 0.001). The pooled proportion of patients achieving sustained low-dose GC remission was 48% (95% CI 27–69, *P* < 0.001) and adverse events occurred in 39% of patients (95% CI 23–44, *P* < 0.001). All the included studies were deemed to be at high risk of bias.

**Conclusion:**

Leflunomide’s utility as a GC-sparing agent is promising but remains to be elucidated in future higher-quality studies. PROSPERO protocol registration CRD42023490373.

Key messages

- Almost half of the patients on leflunomide attained sustained glucocorticoid-free remission.
- Leflunomide’s utility as a glucocorticoid-sparing agent is promising but needs to be validated in future studies.

## Introduction

Giant cell arteritis (GCA) is the most common type of primary vasculitis that occurs in patients ≥50 years of age [1]. GCA preferentially involves temporal arteries and other large vessels, presenting with constitutional symptoms, headaches, jaw claudication, polymyalgia rheumatica and visual disturbances, including permanent vision loss, which is a rheumatologic emergency [2].

The primary treatment for GCA is high-dose glucocorticoids (GCs) for induction, with i.v. methylprednisolone given in cases of vision loss, followed by gradual tapering starting 2 to 4 weeks after GC initiation [3]. Upon tapering GCs, up to one-half of patients will experience a relapse in the first 2 years and will require treatment intensification [4]. Chronic use of GC therapy predisposes patients to major health issues, including osteoporosis, diabetes, infection and cardiovascular disease [5]. Considering these complications, adjunctive GC-sparing agents in the treatment of GCA have been studied, such as methotrexate and tocilizumab [6].

Leflunomide is a dihydroorotate dehydrogenase, with similar downstream cytokine inhibitory effects on IL-6 as tocilizumab [7, 8]. To date, tocilizumab and upadacitinib are the only two US Food and Drug Administration (FDA)-approved adjunctive agents that have demonstrated decreased risk of relapse and reduced GC exposure compared with GC monotherapy [3, 9]. One of the caveats is that tocilizumab suppresses liver synthesis of C-reactive protein (CRP), making it more challenging to monitor disease activity [10]. In this context, leflunomide becomes an attractive alternative agent to consider with its low cost and easy oral administration.

To date, there has been a multitude of studies that report on the use of leflunomide in the treatment GCA [11–18]. All these studies tend to report a positive benefit, although leflunomide’s true effect size remains to be elucidated in a quantitative evidence synthesis with a pooled analysis. The objective of this systematic review is to determine the effectiveness of leflunomide as a GC-sparing agent and its safety profile in the treatment of GCA.

## Methods

This systematic review and meta-analysis were conducted according to the methodological guidelines of the Cochrane Handbook for Systematic Reviews of Interventions (version 6.5) [19]. The protocol and review were reported following the Preferred Reporting Items for Systematic Review and Meta-Analysis Protocols (PRISMA-P) and the PRISMA reporting guidelines, respectively [20]. The protocol for this review was registered (PROSPERO CRD42023490373).

A literature search strategy was created by combining medical subject headings (MeSH) and text keywords for two conceptual categories: GCA and leflunomide (Supplementary Data S1). A publication filter was applied for works published after the GCA classification criteria were introduced on 1 August 1990. No additional restrictions were applied. We searched MEDLINE, Cochrane Central Register of Controlled Trials in the Cochrane Library (CENTRAL) and Embase, as well as study registries such as clinicaltrials.gov. Other grey literature sources, including the American College of Rheumatology Annual Meeting, European League Against Rheumatism Annual Meeting, Web of Science and researchgate.net were also consulted. The search was conducted on 5 May 2024 and updated on 6 January 2025.

### Inclusion and exclusion criteria

We aimed to include randomized controlled trials (RCTs), cohort studies, case-control studies and case series that reported on adult patients with GCA who received leflunomide. The diagnosis and status of the disease were defined according to the ACR classification criteria (version 1990 or version 2022) and the 2021 ACR/Vasculitis Foundation Guideline for the Management of Giant Cell Arteritis and Takayasu Arteritis [21–23]. Refractory disease was defined as inability to induce remission and relapsing disease was defined as recurrence in disease activity after achieving remission [3]. Only studies reporting at least 6 months of follow-up were included. Cross-sectional studies, literature reviews and case reports were excluded. We excluded studies that reported on other types of vasculitides or if any other concomitant therapy was received that was not leflunomide or GC therapy.

### Outcomes

The primary effectiveness outcome was the incidence of patients who attained sustained GC-free remission *vs* GC therapy alone, defined as resolution of clinical symptoms and/or radiological signs and/or biochemical markers as well as the complete discontinuation of GCs. The secondary effectiveness outcome was the mean difference in GC dose in participants on leflunomide *vs* GC therapy alone. Another secondary effectiveness outcome was the incidence of patients who attained sustained low-dose GC remission, as defined by a daily prednisone dose of <5 mg. The primary safety outcome was the incidence of adverse events. Outcomes were preferably assessed after 1 year of follow-up, but as early as 6 months for studies of shorter duration.

### Study selection and data extraction

All search results were exported into Covidence (Melbourne, VIC, Australia) and were independently screened by two reviewers (L.M.Z., J.-P.M.) in the title and abstract phase, with a third reviewer (C.R.) available to resolve remaining discrepancies after discussion. The same process was repeated for full-text screening. A data extraction table was used to collect the relevant information, including characteristics of the study, baseline participant characteristics (age, sex, ethnicity, disease clinical presentation), treatment received (timing/indication for leflunomide, leflunomide dose, GC dose), comparator received (dose of GC and/or other immunosuppressive therapy) and outcomes (clinical and radiologic resolution, GC dose, sustained GC-free remission). Study authors were contacted in the case of missing data via e-mail for a maximum of three attempts.

### Study quality appraisal

We appraised study quality with the respective risk of bias tool for the type of study, including the Risk Of Bias In Non-randomised Studies—of Interventions (ROBINS-I) tool for non-randomised intervention studies and the Risk Of Bias In Non-randomised Studies—of Exposure (ROBINS-E) tool for observational studies [24–26]. Findings were presented in a risk-of-bias table.

### Data synthesis

We performed a meta-analysis using a random-effects model given that the included studies were sufficiently homogeneously reported and tested statistical heterogeneity using the chi-squared test and *I*^2^ statistic. We had initially planned to use the Cochran–Mantel–Haenszel method to evaluate our quantitative binary outcomes, namely proportion of GC-free remission, proportion of low-GC sustained remission and adverse events, however, there were no studies directly comparing leflunomide with GC monotherapy. As a result, we computed the proportions of patients on leflunomide who achieved the primary and secondary outcomes of interest, along with the corresponding 95% CIs, as the effect measure. The results were combined to derive a summary estimate of effect using a random-effects model with a restricted maximum likelihood approach. We had initially planned to use the mean difference as our measure of association for mean reduction in prednisone dose and the inverse variance method for quantitative synthesis. We also conducted subgroup analyses according to disease status (new onset, relapsing and refractory GCA).

### Meta-bias assessment

We generated funnel plots to assess the risk of publication bias and we intended to apply the Egger’s test if the outcome analysis included >10 studies. Outcome reporting bias was evaluated using the Outcome Reporting Bias in Trials (ORBIT) tool [27]. Lastly, the quality of evidence for the outcomes was assessed using the Grading of Recommendations, Assessment, Development, and Evaluation (GRADE) approach [28].

## Results

**Figure 1.**
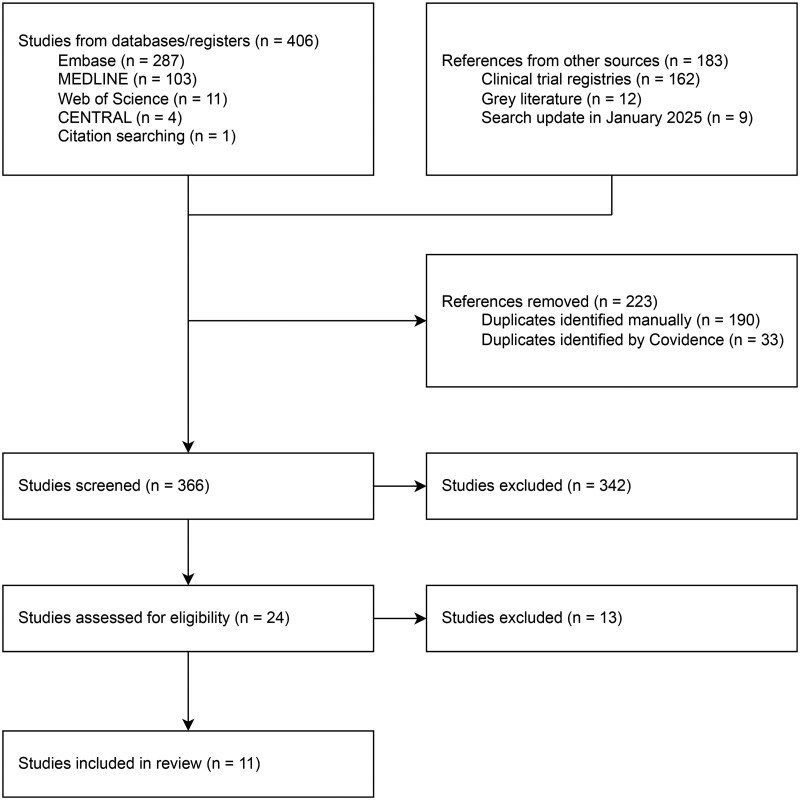
PRISMA flow diagram of included studies

A total of 366 studies were screened, with 11 observational studies included in the final analysis (Fig. 1). Among the 11 studies, there were three prospective cohort studies, five retrospective cohort studies, two case series and one abstract from grey literature. Of the 358 patients pooled for analysis, 231/355 (69%) were females among the studies that reported on participant sex. A total of 233 (65%) patients had new-onset GCA, 83 patients had relapsing GCA (23%) and 19 patients had a refractory GCA (5%). The disease status was not specified for 23 patients (6%). Of the pooled patients, 183/254 (72%) patients presented with constitutional symptoms, 159/212 (75%) with headache and 98/313 (31%) with polymyalgia rheumatica and the median CRP was 27 mg/L [interquartile range (IQR) 24.1–84.3] (Table 1). In seven studies, patients received 10–20 mg daily of leflunomide and in two studies patients received 20 mg daily of leflunomide. The leflunomide dose was not reported in two studies.

**Table 1.** Baseline characteristics of 358 pooled patients treated with leflunomide for GCA

Characteristics	Values
Age, years, median (IQR)	80 (73.8–77.9)
Female, *n* (%)	231 (69)
Disease status, *n* (%)	
New onset	233 (65)
Relapsing	83 (23)
Refractory	19 (5)
Unknown	23 (7)
Constitutional symptoms, *n*/*N* (%)	183/254 (72)
Polymyalgia rheumatica, *n*/*N* (%)	98/313 (31)
Headache, *n*/*N* (%)	159/212 (75)
Jaw claudication, *n*/*N* (%)	89/201 (44)
Visual symptoms, *n*/*N* (%)	45/206 (22)
Visual loss, *n*/*N* (%)	24/206 (12)
Stroke, *n*/*N* (%)	7/176 (4)
CRP, mg/L, median (IQR)	27 (24.1–84.3)
Radiologic findings of large vessel vasculitis, *n*/*N* (%)	54/92 (59)
Biopsy-proven disease, *n*/*N* (%)	32/117 (27)

All numbers are reported as n/group N (%) unless otherwise specified. IQR: interquartile range, CRP: C-reactive protein.

**Figure 2.**
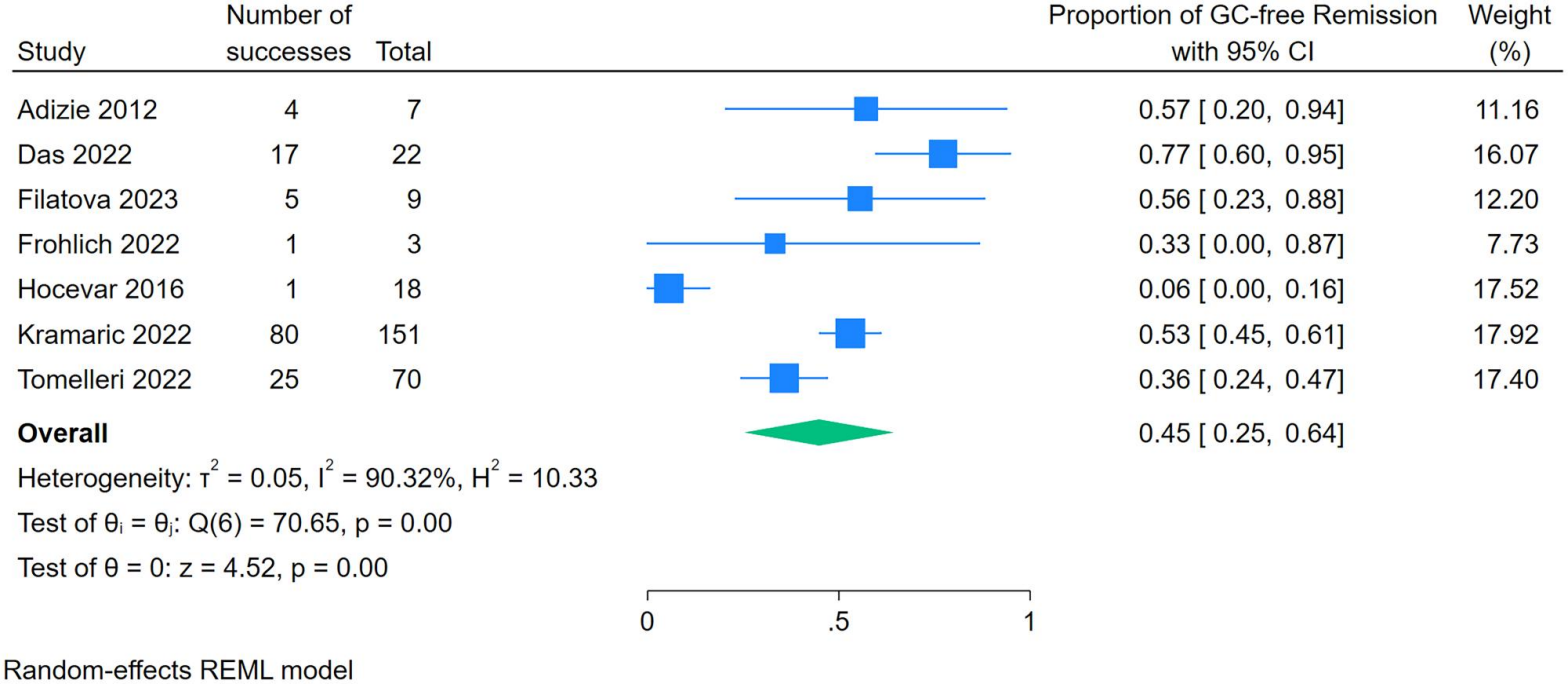
Meta-analysis of proportions for the effectiveness of leflunomide to achieve GC-free remission in patients with GCA

**Figure 3.**
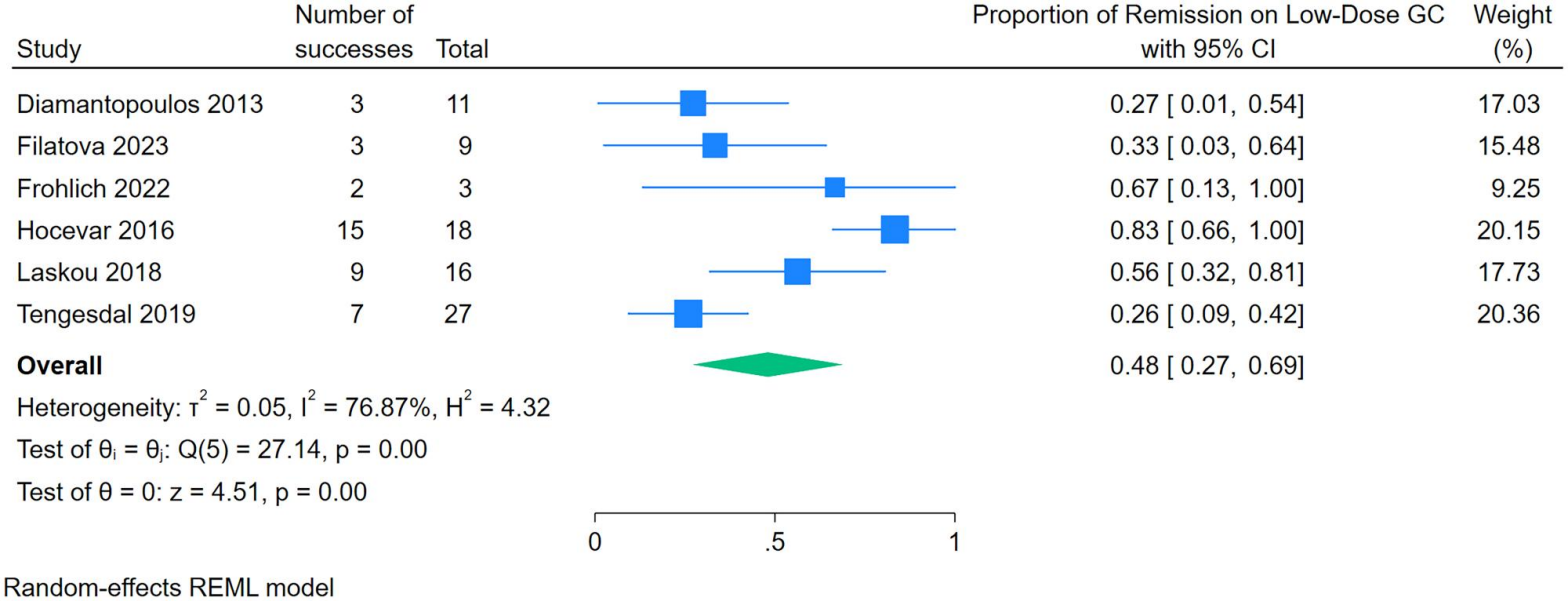
Meta-analysis of proportions for the effectiveness of leflunomide to achieve low-dose GC remission in patients with GCA

**Figure 4.**
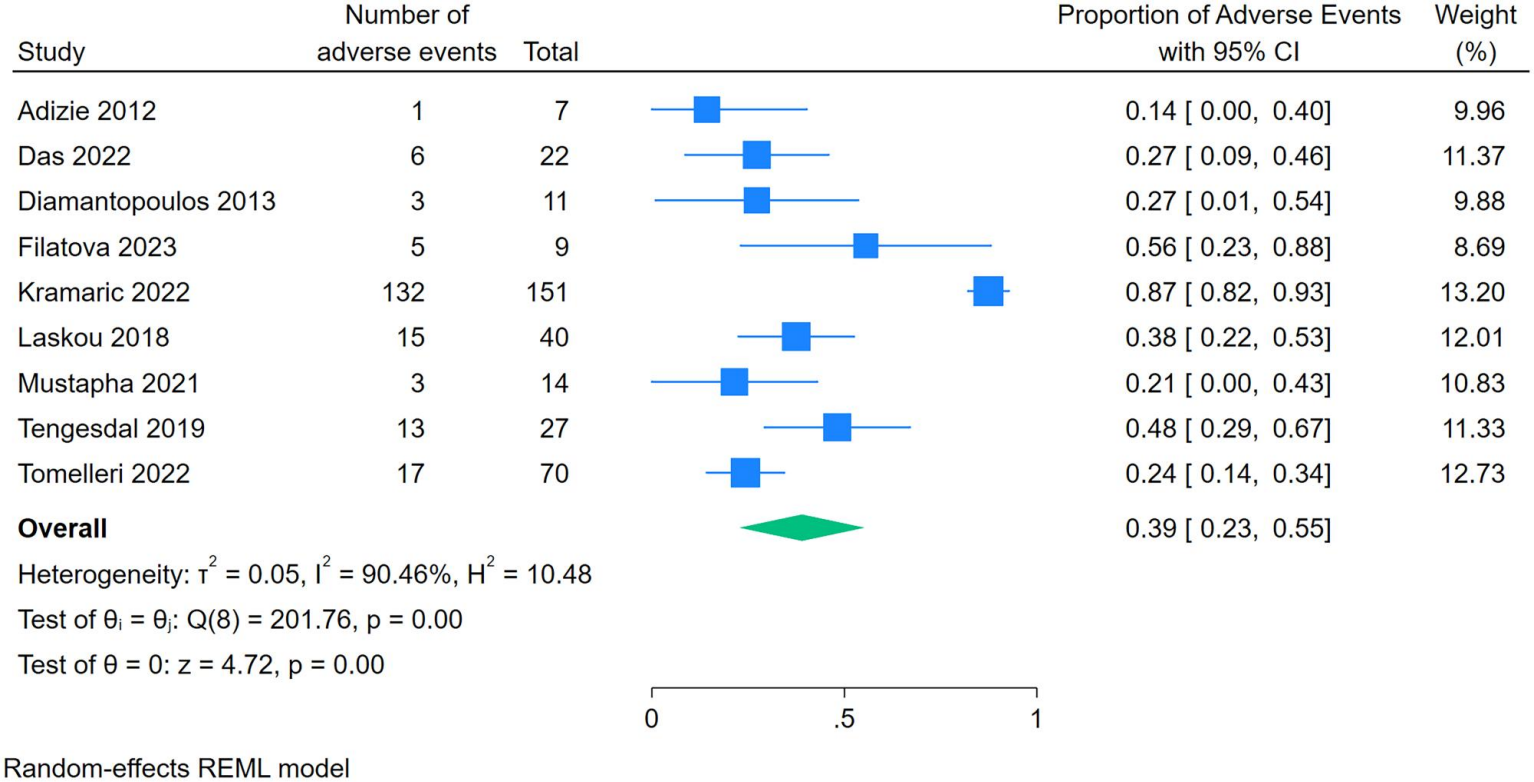
Meta-analysis of proportions for adverse events of leflunomide in the treatment of GCA

The pooled proportion of patients achieving GC-free remission with leflunomide was 45% (95% CI 0.25–0.64, *P* < 0.001, seven studies) (Fig. 2). Substantial statistical heterogeneity was observed (*I*^2^ = 90.3%, *Q* = 70.7, *P* < 0.001). Meanwhile, the pooled proportion of patients achieving low-dose GC remission was 48% (95% CI 0.27–0.69, *P* < 0.001, six studies) (Fig. 3). We were unable to compare our primary and secondary effectiveness outcome with a GC-only comparator group, as no included studies reported on a GC-only comparator group. We were unable to analyse the mean reduction in prednisone dose due to heterogeneous reporting in the included studies. A subgroup analysis of leflunomide for new-onset GCA showed that 60% of patients achieved GC-free remission (95% CI 0.39–0.81, *P* < 0.001, three studies) (Supplementary Fig. S1). We were unable to perform subgroup analyses on relapsing or refractory GCA due to limited reporting on outcomes by subgroups. Adverse events occurred in 39% of patients (95% CI 0.23–0.44, *P* < 0.001, nine studies) (Fig. 4). The most reported adverse events included infection, gastrointestinal symptoms, liver function test abnormalities and cardiovascular outcomes (Table 2). Four studies reported on leflunomide discontinuation specifically due to adverse events: Kramaric *et al.* [15] and Laskou *et al.* [29] provided specific proportions, 41/151 (27%) and 3/40 (8%), respectively, while in the studies by Tengesdal *et al.* [17] and Tomelleri *et al.* [18], all patients experiencing adverse events discontinued leflunomide treatment. All other included studies did not clarify if adverse events necessitated leflunomide discontinuation. Funnel plots were slightly asymmetrical, which suggested the presence of publication bias as well as possible poor methodological quality in the reporting of outcomes (Supplementary Fig. S2). All the included studies were deemed to be at serious risk of bias per the ROBINS-I tool (Supplementary Table S1). We assessed the certainty of evidence using the GRADE approach, even though the included studies lacked a comparator group. Due to study design limitations and the potential for confounding, the certainty of evidence was rated as low.

**Table 2.** Adverse events in 358 pooled patients treated with leflunomide for GCA

Adverse events	Values, *n*/*N* (%)
Infection	34/273 (12)
Cardiovascular outcomes (CAD, HTN, TIA)	11/252 (4)
Liver function test abnormalities	7/200 (4)
Gastrointestinal symptoms (peptic ulcer disease, diarrhoea)	35/279 (13)
Polyneuropathy	2/23 (9)
Allergic reaction	3/70 (4)
Lymphoma	1/70 (1)
Hair loss	46/221 (21)
Weight loss	12/151 (8)
Malaise	4/27 (15)
Cough	2/70 (3)
Skin bruising	79/151 (52)
GC-induced diabetes	41/151 (27)
GC-induced myopathy	39/151 (26)
Osteoporotic fractures	8/151 (5)
Cataracts	18/151 (12)

CAD: coronary artery disease, HTN: hypertension; TIA: transient ischaemic attack.

We also assessed outcome reporting bias using the ORBIT approach (Supplementary Table S2) [27]. In four of the 11 included studies, the primary outcome was not reported, and it was unclear whether it was measured. Two of the 11 studies did not measure adverse events.

## Discussion

Our systematic review sought to evaluate the effectiveness and safety of leflunomide as a GC-sparing agent in the treatment of GCA. Our study found that almost half of patients receiving leflunomide were able to achieve GC-free remission at 1 year. However, our conclusions were substantially limited by the heterogeneity and high risk of bias in our included observational studies. Nonetheless, this is a notable observation, as in most cases of GCA, following induction of remission with GC therapy, the course of GC therapy tapering is often met with the challenge of disease relapse. As few as 18% of patients will sustain GC-free remission after a 52-week taper (as many as 82% of patients will experience a relapse on GC monotherapy), and these patients will have a greater cumulative prednisone dose compared with non-relapsing patients, thereby subjecting them to a greater burden of risk of GC-associated health complications, including osteoporosis, hypertension, diabetes and infection [30]. Leflunomide therefore presents as an interesting GC-sparing option that warrants further investigation. Currently, tocilizumab and upadacitinib are the only FDA-approved adjunctive immunosuppressive therapy for the treatment of GCA [3, 9]. The Trial of Tocilizumab in Giant Cell Arteritis study showed a sustained remission rate of 56% at 52 weeks in patients receiving weekly tocilizumab doses and 53% in those receiving doses every 2 weeks [30]. The SELECT-GCA study (NCT03725202) also demonstrated similar results in patients receiving a daily dose of 15 mg upadacitinib, with a sustained remission of 46% on a 26-week GC taper [9]. While a direct comparison cannot be made, these remission rates are similar to the 45% sustained remission observed with leflunomide in our study. Additionally, leflunomide is less expensive and offers a simpler mode of administration in pill form compared with tocilizumab. An added advantage of leflunomide is that it does not directly inhibit CRP synthesis, unlike tocilizumab, thereby allowing clinicians to continue using this inflammatory marker to assess disease activity. Unfortunately, we were unable to explore in our study the dose-dependent effect of leflunomide on treatment outcomes due to heterogeneous reporting in the included studies.

The safety profile of leflunomide in our study was comparable to what has already been reported in the literature. The main adverse events included gastrointestinal complaints, liver enzyme abnormalities, infection and hair loss, although this was mostly studied in the treatment of RA [31–33]. Of note, the adverse events reported in the included studies were while patients were often receiving both leflunomide and GC therapy. No included study differentiated between GC-related and leflunomide-related adverse events.

A recent systematic review by Narváez *et al.* [34] examined the use of leflunomide in the treatment of large vessel vasculitis, including seven observational studies. They reported a partial remission rate of 60%, and 53% of patients were able to completely discontinue GC therapy, which aligns with our findings, although their definition of partial or complete remission was defined by the investigators of the respective studies. Our search strategy included grey literature, and we included studies reporting on a minimum of 6 months follow-up compared with 12 weeks in the study of Narváez *et al*. [34]. Furthermore, their definition of complete or partial remission was defined according to study investigators, whereas we delineated specific objective measures for our primary outcome. Although their review differed in terms of inclusion criteria and outcome measures, we found similar effectiveness estimates, supporting our results. But again, these similarities should be interpreted with caution in the context of the limitations of observational data.

Our study has several strengths. The rigorous search strategy developed in collaboration with systematic review experts ensures a comprehensive and methodologically sound approach to identifying relevant studies. In addition, we included grey literature, reducing the risk of publication bias and capturing relevant studies that may not have been included in traditional peer-reviewed sources. Another key strength is our focus on the outcome of complete GC discontinuation, a sought-after target in the treatment of GCA and a key endpoint in clinical trials. This makes our findings highly relevant to both clinical practice and future research. Furthermore, our review synthesizes real-world evidence from observational studies, providing valuable insights into the effectiveness of leflunomide in GCA, which can be directly applied to clinical settings.

Our study also has limitations. We only found and included non-randomized observational studies, which introduces several potential biases. First, there is significant heterogeneity in the reporting of clinical, laboratory and radiological findings across the included studies. This variability can limit the comparability of results and introduce inconsistencies in the interpretation of outcomes. Additionally, the observational study design inherently carries the risk of confounding factors, as these studies lack randomization, meaning that only associations, rather than causal relationships, can be established. Finally, differences in inclusion criteria and outcome measures across the studies further limit the ability to draw firm conclusions or generalize the findings universally. These limitations should be considered when interpreting the results of our review.

Our systematic review of observational data suggests leflunomide’s potential utility for achieving GC-free and low-dose GC remission in GCA. Given the substantial heterogeneity and risk of bias, these findings should be interpreted with caution and cannot be taken as definitive evidence of effectiveness or safety. High-quality, large-scale RCTs are warranted to more conclusively demonstrate the effectiveness and safety profile of leflunomide in the treatment of GCA.

## Supplementary Material

rkaf128_Supplementary_Data

## Data Availability

No new data were generated or analysed in support of this article.
