# Supplementary material for: The effectiveness and safety of leflunomide in the treatment of giant cell arteritis: a systematic review and meta-analysis
Source: Rheumatol Adv Pract. 2025 Nov 7;9(4):rkaf128. doi: 10.1093/rap/rkaf128 (PMC12657133; doi:10.1093/rap/rkaf128)

**Supplementary Material**

Supplementary Data S1: MEDLINE (OVID) search strategy

Ovid MEDLINE(R) ALL <1946 to May 18, 2023>

1 Giant Cell Arteritis/

2 exp vasculitis/

3 temporal arteritis.tw.

4 (giant cell adj3 arteritis).tw.

5 1 or 2 or 3 or 4

6 exp Leflunomide/

7 leflunomide.tw.

8 6 or 7

9 5 and 8

Supplementary Table S1: ROBINS-I risk of bias assessment of the 11 included studies
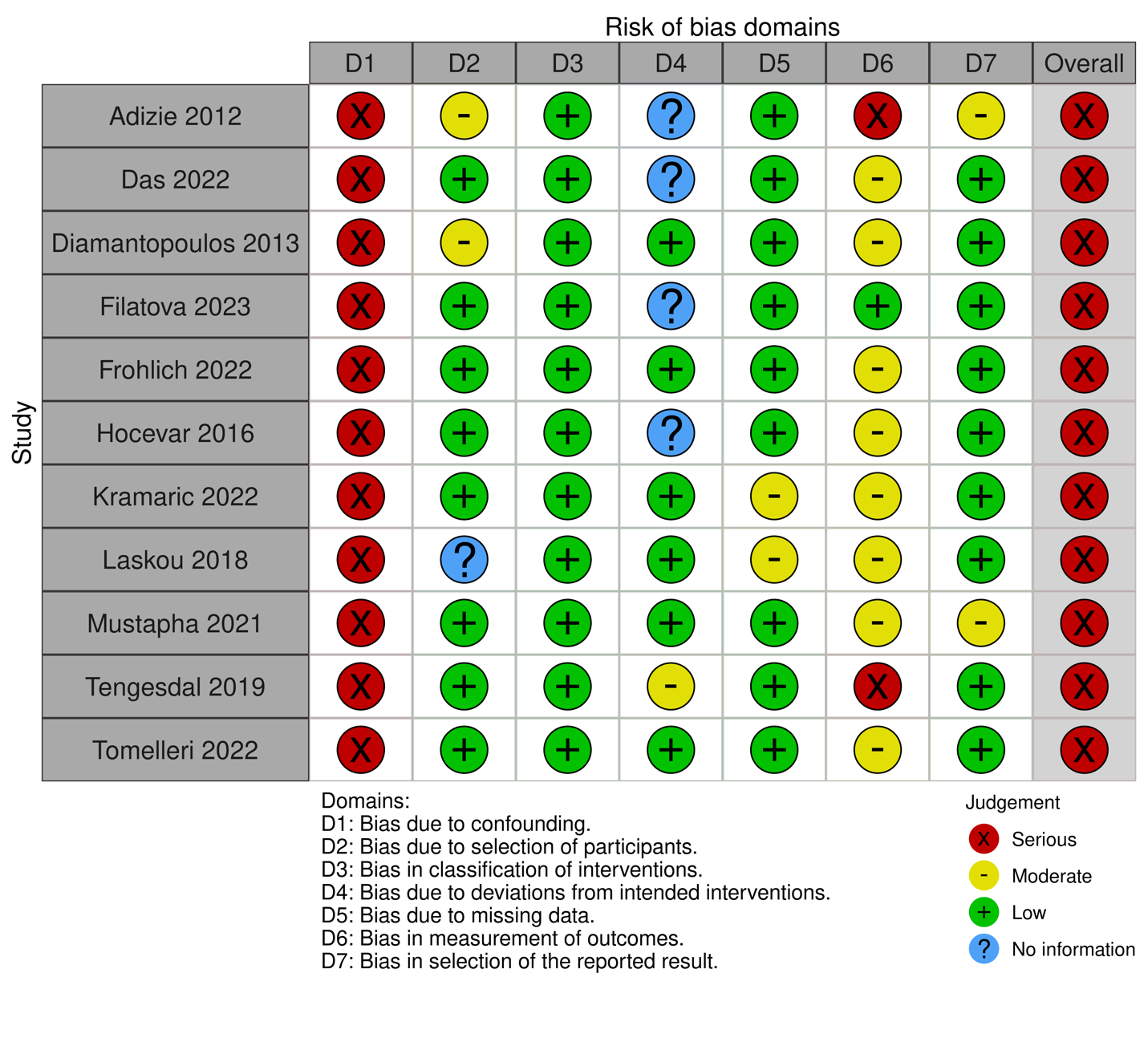


Supplementary Table S2: Outcome reporting bias in trials (ORBIT) of the 11 included studies

| Study ID (author, date of publication) | Review primary outcomes | Review harm outcomes |
| --- | --- | --- |
|  | GC-free remission | Adverse events |
| Adizie 2012 | 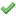 | 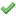 |
| Das 2022 | 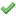 | 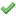 |
| Diamantopoulos 2013 | 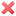 F | 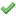 |
| Filatova 2023 | 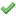 | 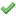 |
| Frohlich 2022 | 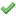 | 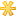 |
| Hocevar 2016 | 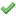 | 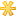 |
| Kramaric 2022 | 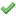 | 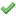 |
| Laskou 2018 | 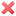 E | 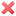 S1 |
| Mustapha 2021 | 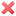 F | 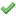 |
| Tengesdal 2019 | 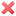 F | 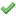 |
| Tomelleri 2022 | 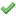 | 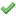 |


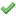
: Outcome was fully and completely reported.


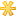
: Outcome was not measured.


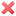
: Outcome was not reported, but unclear if whether it was measured.

E: The benefit outcome was measured. Judgment says outcome likely to have been analysed but not reported because of non-significant results.

F: The benefit outcome was measured. Judgment says outcome unlikely to have been analysed.

S1: The harm outcome was not explicitly mentioned. Judgment says likely measured and compared, but only pooled adverse events were reported.

Supplementary Figure S1: Subgroup analysis of proportions for the effectiveness of leflunomide to achieve GC-free remission in patients with new onset giant cell arteritis


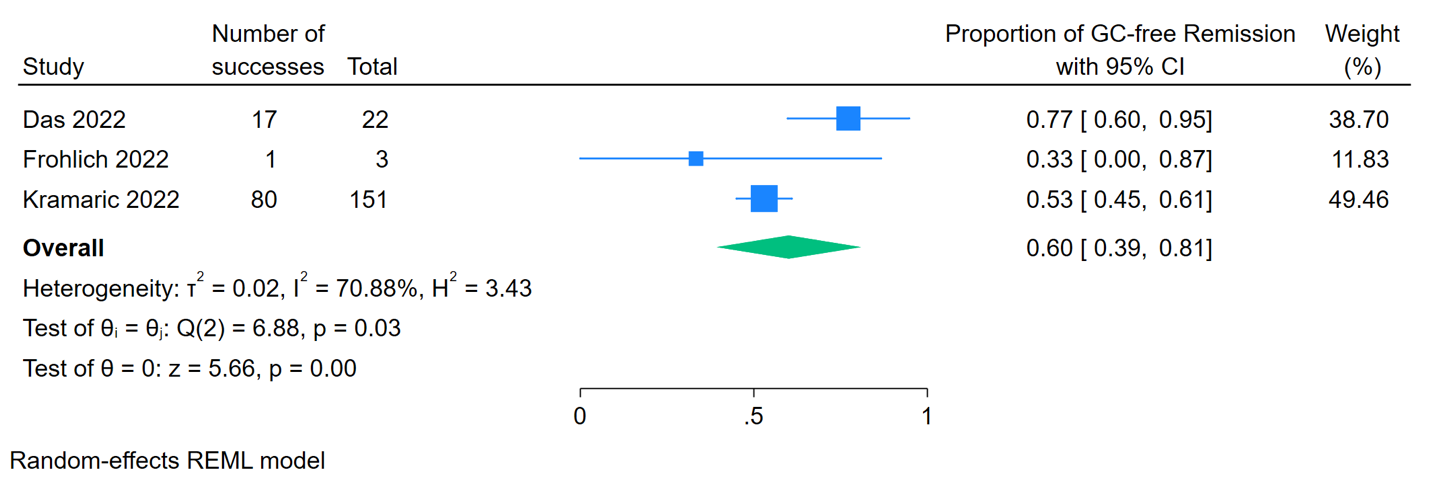


Supplementary Figure S2: Funnel plot of the meta-analysis for the outcome of GC-free remission in the treatment of giant cell arteritis with leflunomide (7 studies)


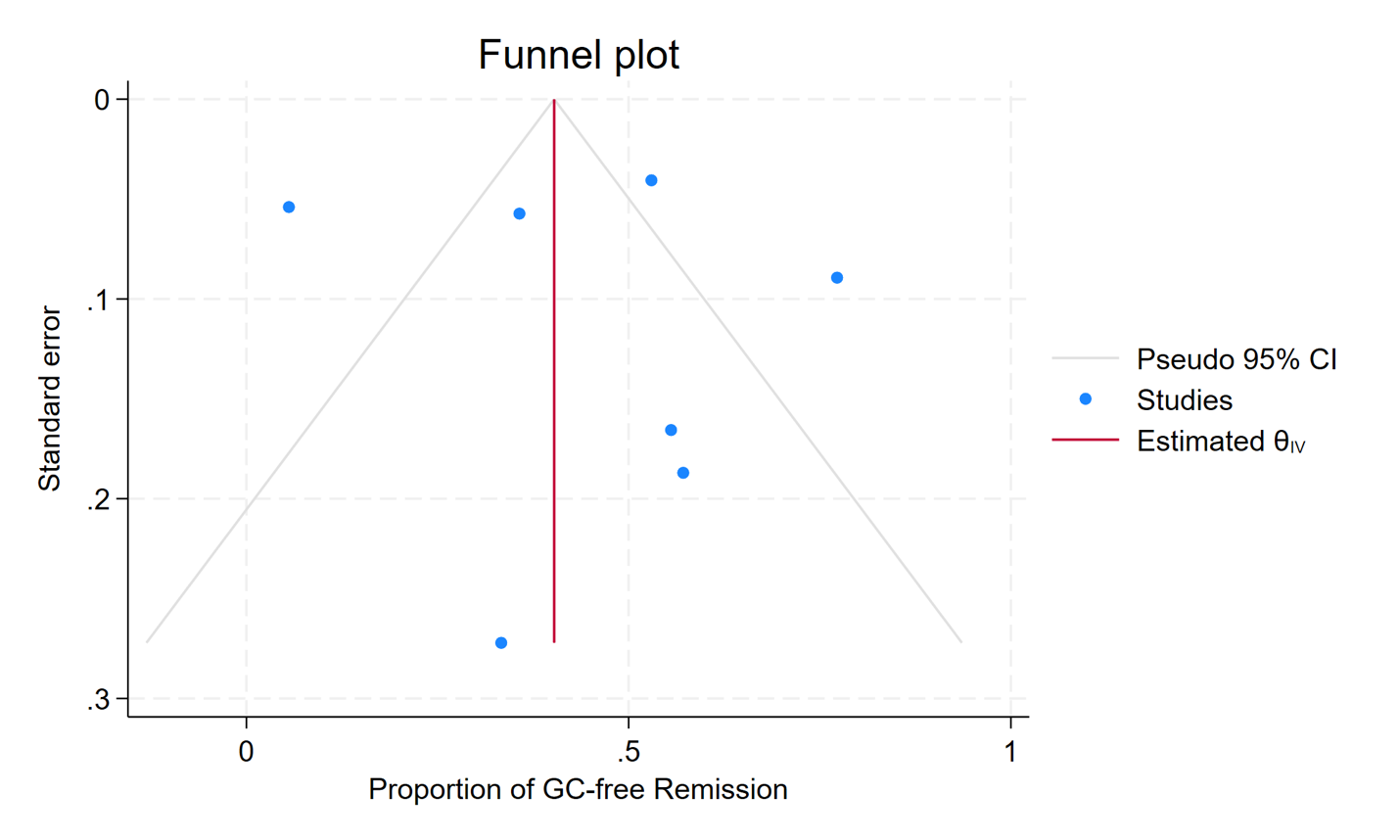

Supplement: rkaf128_Supplementary_Data [file rkaf128_supplementary_data.docx]
